# Clinical Efficacy of the Neutralizing Antibody Therapy Sotrovimab in Patients with SARS-CoV-2 Omicron BA.1 and BA.2 Subvariant Infections

**DOI:** 10.3390/v15061300

**Published:** 2023-05-31

**Authors:** Naoyuki Miyashita, Yasushi Nakamori, Makoto Ogata, Naoki Fukuda, Akihisa Yamura, Yoshihisa Ishiura, Tomoki Ito

**Affiliations:** 1 Division of Respiratory Medicine, Infectious Disease and Allergology, First Department of Internal Medicine, Kansai Medical University, 2-3-1 Shin-machi, Hirakata 573-1191, Japan; ogatam@hirakata.kmu.ac.jp (M.O.); fukudana@hirakata.kmu.ac.jp (N.F.); yamuraak@hirakata.kmu.ac.jp (A.Y.); itot@hirakata.kmu.ac.jp (T.I.); 2 Department of Emergency Medicine, Kansai Medical University Medical Center, Moriguchi 570-8507, Japan; 99nakamori@gmail.com; 3 Division of Respiratory Medicine, Oncology and Allergology, First Department of Internal Medicine, Kansai Medical University Medical Center, Moriguchi 570-8507, Japan; ishiuray@takii.kmu.ac.jp

**Keywords:** sotrovimab, monoclonal antibody, COVID-19, Omicron variant, SARS-CoV-2

## Abstract

Sotrovimab, an antibody active against severe acute respiratory syndrome coronavirus 2 that neutralizes antibodies, reduced the risk of COVID-19-related hospitalization or death in studies conducted before the emergence of the Omicron variant. The objective of this study is to evaluate the clinical efficacy of sotrovimab in patients with mild to moderate COVID-19 Omicron BA.1 and BA.2 subvariant infections using a propensity score matching method. The propensity score-matched cohort study population was derived from patients who received sotrovimab. We derived a comparator group from an age- and sex-matched population who were recuperating in a medical facility after COVID-19 infection or from elderly person entrance facilities during the same period who were eligible for but did not receive sotrovimab treatment. In total, 642 patients in the BA.1 subvariant group and 202 in the BA.2 subvariant group and matched individuals were analyzed. The outcome was the requirement for oxygen therapy. In the treatment group, 26 patients with the BA.1 subvariant and 8 patients with the BA.2 subvariant received oxygen therapy. The administration of oxygen therapy was significantly lower in the treatment group than in the control group (BA.1 subvariant group, 4.0% vs. 8.7%, *p* = 0.0008; BA.2 subvariant group, 4.0% vs. 9.9%, *p* = 0.0296). All these patients were admitted to our hospitals and received additional therapy and then recovered. No deaths were observed in either group. Our results demonstrate that the sotrovimab antibody treatment may be associated with a reduction in the requirement for oxygen therapy among high-risk patients with mild to moderate COVID-19 Omicron BA.1 and BA.2 subvariants.

## 1. Introduction

Sotrovimab is a monoclonal antibody active against severe acute respiratory syndrome coronavirus 2 (SARS-CoV-2) that has been indicated for patients over 12 years of age who do not require oxygen therapy but have an increased risk of hospitalization or death. In studies performed before the emergence of the Omicron (B.1.1.529) BA.1 subvariant, sotrovimab reduced emergency department visits, hospitalization, death, and progression to severe or critical respiratory COVID-19 [1,2,3,4,5]. A randomized controlled trial including 1057 non-hospitalized patients with symptomatic, mild to moderate COVID-19 demonstrated that sotrovimab significantly reduced the risk of a composite end point of all-cause hospitalization or death until day 29 (sotrovimab 1% vs. placebo 6%, adjusted relative risk (RR) 0.21 (95% confidence interval [CI], 0.09 to 0.50)) [1,2]. In addition, secondary outcomes were significantly in favor of sotrovimab, including reduced emergency department visits, hospitalization, death, and progression to severe or critical respiratory COVID-19 [1,2]. S309 (the precursor of sotrovimab), which has been shown to have a lower neutralizing activity against the BA.1 subvariant than against the ancestral strain and other variants of concern [6,7], had an even lower neutralizing activity against the BA.2 subvariant [8,9]. Results from in vitro pseudovirus assays showed that sotrovimab neutralized the BA.1 and BA.2 subvariants with a 2.7-fold and 16.0-fold change in the half maximal inhibitory concentration (IC_50_) relative to the wild-type, respectively [10,11].

Despite losses in the neutralization potency in cell cultures, S309 treatment reduced BA.2 lung infection in susceptible mice that express human ACE2 (K18-hACE2) in prophylactic and therapeutic settings [12]. Correlation analyses between in vitro neutralizing activity and reductions in viral burden in K18-hACE2 or human FcγR transgenic mice suggested that S309 had different mechanisms of protection against the Omicron variants, with S309 utilizing Fc effector function (antibody-dependent cellular cytotoxicity (ADCC) activity and antibody-dependent cell-mediated phagocytosis (ADCP) activity) interactions [12]. Bruel et al. evaluated the neutralization and ADCC activities of six therapeutic monoclonal antibodies against BA.2, and sotrovimab was the most efficient at eliciting ADCC [13]. Thus, sotrovimab may be clinically useful against the COVID-19 Omicron BA.2 subvariant.

The purpose of this study is to evaluate the clinical efficacy of sotrovimab in patients with the COVID-19 Omicron BA.2 subvariant and to compare these results with the BA.1 subvariant using a propensity score matching method. Our results demonstrate that the sotrovimab antibody treatment may be associated with a reduction in the requirement for oxygen therapy among high-risk patients with mild to moderate COVID-19 Omicron BA.1 and BA.2 subvariants.

## 2. Subjects and Methods

### 2.1. COVID-19 Patients

The present study was conducted at five institutions (Kansai Medical University Hospital, Kansai Medical University Medical Center, Kansai Medical University Kori Hospital, Kansai Medical University Kuzuha Hospital, and Kansai Medical University Temmabashi General Clinic) between December 2021 and July 2022, and assessed patients with mild to moderate COVID-19 BA.1 and BA.2 subvariants. COVID-19 was diagnosed using a positive reverse transcription polymerase chain reaction (RT-PCR) test from nasopharyngeal swab specimens in accordance with the protocol recommended by the National Institute of Infectious Diseases, Japan. The identification of the SARS-CoV-2 variants was performed by the Sanger sequencing of the Spike coding gene using an ABI 3500 analyzer (Applied Biosystems, Waltham, MA, USA) [14].

### 2.2. Inclusion Criteria of Sotrovimab Treatment

The following inclusion criteria, based on the package insert, were used to administer sotrovimab: (1) positive SARS-CoV-2 antigen or RT-PCR tests on specimens taken within 7 days prior to enrollment, (2) compatible symptoms of onset of SARS-CoV-2 infection no more than 5 days before administration, (3) oxygen saturation level with room air of 94% or more, and (4) the patient had at least one of the following risk factors: age ≥55 years old, diabetes for which medication was warranted, obesity with body mass index ≥30 kg/m^2^, chronic kidney disease (estimated glomerular filtration rate, <60 mL per minute per 1.73 m^2^ of body surface area), congestive heart failure (New York Heart Association class II, III, or IV), chronic obstructive pulmonary disease, and moderate to severe asthma with a need for an inhaler or who used oral steroids within 1 year prior to enrollment. 

### 2.3. Treatment Group and Control Group

The propensity score-matched cohort study population was derived from patients with mild to moderate COVID-19 who received sotrovimab treatment. Sotrovimab recipients were consulted in our hospitals from the Follow-up Center in Osaka prefecture. An intravenous infusion of sotrovimab was used in the outpatient department except for patients with a low oxygen saturation level. In addition, we visited a COVID-19 accommodation medical facility (hotel recuperation) and elderly person entrance facilities to deliver an intravenous drip at the request of the health center. The treatment group received a dose of sotrovimab 500 mg intravenously.

We derived a comparator group from an age- and sex-matched population who were recuperating in a COVID-19 accommodation medical facility or elderly person entrance facilities by identifying patients with a positive RT-PCR result for SARS-CoV-2 during the same period who were eligible for but did not receive sotrovimab treatment. In the BA.1 subvariant group, we made propensity score matches from among 676 patients who received sotrovimab treatment and 1321 patients without treatment, and 642 matched individuals in each group were analyzed. In the BA.2 subvariant group, we made propensity score matches from among 223 patients who received sotrovimab treatment and 673 patients without treatment, and 202 matched individuals in each group were analyzed. Informed consent was obtained from all patients, and the study protocol was approved by the Ethics Committee of Kansai Medical University (approval number 2020319).

The end point was a requirement for oxygen therapy (either nasal canula, high-flow nasal cannula (HFNC) oxygenation, or mechanical ventilation). We used a Wilcoxon rank sum test for continuous variables, and Fisher’s exact test for categorical variables.

## 3. Results

### 3.1. Patient Characteristics in the BA.1 Subvariant Group

The characteristics of the patients in the BA.1 subvariant group are shown in Table 1. The treated patients received sotrovimab as outpatients (n = 129), as inpatients (n = 63), at an accommodation medical facility (n = 382), and at *elderly person entrance facilities* (n = 68). The most common risk factor was age ≥50 years old (n = 389), followed by obesity with a body mass index ≥30 kg/m^2^ (n = 121), diabetes (n = 108), chronic obstructive pulmonary disease (n = 86), chronic kidney disease (n = 62), moderate to severe asthma (n = 56), and congestive heart failure (n = 30). No patients with immunocompromising conditions or who used immunocompromising medications were observed in either group. Five hundred and fifty-one patients were vaccinated (BNT162b2 or mRNA-1273) against SARS-CoV-2 at least once in the treatment group. Median time from symptom onset to treatment and to informed consent for treatment in the control group was 3 days. No significant differences with risk factors, vaccination, or laboratory findings were observed between the treatment and control groups.

### 3.2. Efficacy of Sotrovimab in the BA.1 Subvariant Group

Twenty-six patients in the treatment group and fifty-six patients in the control group received oxygen therapy (Table 2). The administration of oxygen therapy was significantly lower in the treatment group than the control group (*p* = 0.0008). In the treatment group, 20 patients received a nasal canula, 4 patients received HFNC, and 2 patients received mechanical ventilation. All these patients were admitted to our hospitals and received additional therapy (remdesivir, baricitinib, and/or corticosteroids) and recovered. No deaths were observed in either group.

### 3.3. Patient Characteristics in the BA.2 Subvariant Group

The characteristics of the patients in the BA.2 subvariant group are shown in Table 3. The treated patients received sotrovimab as outpatients (n = 5), as inpatients (n = 16), and at an accommodation medical facility (n = 181). The most common risk factor was age ≥50 years old (n = 109), followed by obesity with a body mass index ≥30 kg/m^2^ (n = 36), diabetes (n = 32), chronic obstructive pulmonary disease (n = 19), moderate to severe asthma (n = 18), chronic kidney disease (n = 17), and congestive heart failure (n = 9). No patients with immunocompromising conditions or who used immunocompromising medications were observed in either group. One hundred and forty patients were vaccinated (BNT162b2 or mRNA-1273) against SARS-CoV-2 at least once in the treatment group. The median time from symptom onset to treatment and to informed consent of treatment in the control group was 3 days. No significant differences in risk factors, vaccination, or laboratory findings were observed between the treatment and control groups.

### 3.4. Efficacy of Sotrovimab in the BA.2 Subvariant Group

Eight patients in the treatment group and twenty patients in the control group received oxygen therapy (Table 4). The administration of oxygen therapy was significantly lower in the treatment group than the control group (*p* = 0.0296). In the treatment group, six patients received a nasal canula, and two patients received HFNC. Two patients in the control group received mechanical ventilation. All these patients were admitted to our hospitals and received additional therapy and recovered. No deaths were observed in either group.

## 4. Discussion

Before the emergence of the Omicron variant, in a propensity score-matched cohort study and randomized comparative effectiveness trial, sotrovimab treatment was associated with a reduced risk of hospitalization or death in non-hospitalized patients with mild to moderate COVID-19 caused by the Delta variant [3]. An observational cross-sectional study using hospitalized COVID-19 patients supported the use of sotrovimab for early treatment in patients with mild to moderate COVID-19 at a high risk of disease progression [5]. In contrast, another double-blind, placebo-controlled, randomized controlled trial demonstrated that sotrovimab did not show efficacy over placebo for improving clinical outcomes among adults hospitalized with COVID-19 [13]. At day 5, the sotrovimab group had significantly higher odds of more favorable outcomes than the placebo group on either the pulmonary scale (adjusted odds ratio (OR) sotrovimab 1.07 (95% CI 0.74–1.56)) or the pulmonary-plus complications scale (OR 1.08 (95% CI 0.74–1.58)). Thirteen (7%) patients in the placebo group and fourteen (8%) in the sotrovimab group died up to day 90 [15].

During the SARS-CoV-2 Delta and Omicron BA.1 and BA.2 subvariant waves in the US, the clinical effectiveness of sotrovimab in reducing the risk of 30-day all-cause hospitalization and/or mortality was evaluated [16]. The sotrovimab cohort showed a 55% lower risk of 30-day hospitalization or mortality and an 85% lower risk of 30-day mortality [16]. The RR reduction for 30-day hospitalization or mortality in the sotrovimab cohort was maintained monthly, ranging from 46% to 71% compared with the no monoclonal antibody cohort. The RR reduction for 30-day hospitalization or mortality in the sotrovimab cohort in March 2023 (the prevalence of BA.2 variant and sublineages was approximately 50%) was 59% [16]. During the SARS-CoV-2 Omicron BA.1 and BA.2 waves in England, sotrovimab was associated with a lower risk of severe outcomes of COVID-19 than treatment with molnupiravir, which is active against the BA.1 and BA.2 subvariants [17]. In the BA.1-subvariant-predominant period, 32 (0.96%) of 3331 patients treated with sotrovimab were admitted to hospital or died from COVID-19 during 28 days of follow-up after the start of treatment. Of these 32 patients, 7 (0.21%) died of COVID-19 during the 28 days of follow-up. In the BA.2-subvariant-predominant period, 57 (0.95%) of 5979 patients treated with sotrovimab were admitted to hospital or died from COVID-19 during 28 days of follow-up after the start of treatment. Of these 57 patients, 9 (0.15%) died of COVID-19 during the 28 days of follow-up. The clinical efficacy of sotrovimab and molnupiravir were identical between the BA.1- and BA.2-predominant periods [17].

Although there was a relatively small number of BA.2-infected patients, sotrovimab was associated with a lower incidence of COVID-19-related hospitalization or death among very-high-risk patients with mild to moderate COVID-19 related to the BA.1 and BA.2 subvariants [18]. In contrast, Zaqout et al. demonstrated that sotrovimab treatment was not associated with a reduced risk of COVID-19 severity in the period dominated by the BA.2 subvariant in Qatar [19]. The adjusted OR of disease progression to severe, critical, or fatal COVID-19 comparing the sotrovimab treatment group with the control group was 2.67 (95% CI 0.60–11.91). In the analysis including only the subgroup of patients at higher risk of severe forms of COVID-19, the adjusted OR was 0.65 (95% CI 0.17–2.48). Our study demonstrated that the clinical efficacy of sotrovimab was identical between the BA.1 and BA.2 subvariant groups when the outcome was a requirement for oxygen therapy. The administration of oxygen therapy was significantly lower in the treatment group than the control group (BA.1 subvariant group, 4.0% vs. 8.7%, *p* = 0.0008; BA.2 subvariant group, 4.0% vs. 9.9%, *p* = 0.0296). In addition to the oxygen therapy requirement, we also assessed the recovery time that was prescribed with the disappearance of symptoms (fever, sore throat, and/or nasal symptoms). The median recovery time was significantly lower in the treatment group than the control group (BA.1 subvariant group, 6.1 days vs. 7.9 days, *p* = 0.0092; BA.2 subvariant group, 6.5 days vs. 8.3 days, *p* = 0.0156). The conclusion of the studies is based on the number of patients who progressed to severe in Qatar’s study and who received oxygen therapy and death in our study between the sotrovimab and control groups. However, the number of patients who progressed to severe or qualified oxygen therapy is very small [19]. A small sample size may have influenced the divergence results.

In addition to in vitro pseudovirus assays (BA.1 (2.7-fold) and BA.2 (16.0-fold)), in vitro live authentic virus assays showed 3.8-fold and 15.7-fold changes in IC_50_ against the BA.1 and BA.2 subvariants relative to the wild-type, respectively [10,11]. However, sotrovimab has different mechanisms of protection against the Omicron variants utilizing the Fc effector function, ADCC, and ADCP activities [10]. The neutralization dose did not correlate with ADCC activity [11]. These activities of sotrovimab may have played a role in clinical effectiveness against the BA.2 subvariant. To elucidate the discrepancy in the in vitro and in vivo results, a large sample size study targeting patients with Omicron sublineages is needed. As of June 2023, the Omicron XBB subvariant is the mainstream. In vitro pseudovirus assays showed 6.5-fold and 11.3-fold changes in IC_50_ against the XBB.1 and XBB.1.5 subvariants relative to the wild-type, respectively, and in vitro live authentic virus assays showed a 33.3-fold change in IC_50_ against the XBB.1.5 subvariant relative to the wild-type [10,11]. Sotrovimab efficiently promoted the ADCC of cells expressing Wu-D614, BA.2, BQ.1.1, or XBB.1 S in a concentration- and Fc-dependent manner [20].

The United States Food and Drug Administration (FDA) deauthorized sotrovimab for COVID-19 on rolling basis across states with above 50% Omicron BA.2 prevalence and, on 5 April 2022, deauthorized it across the country because the authorized dose of sotrovimab is unlikely to be effective against the BA.2 subvariant [18]. Healthcare providers should use other approved or authorized products as they choose appropriate treatment options for patients.

Our study had several limitations. First, we evaluated the oxygen requirement, recovery time, and death. We did not evaluate viral loads. Second, in both treatment cohorts, a higher proportion of the population was vaccinated than in the control group. Thus, it may be hard to distinguish between the vaccine and sotrovimab, which boosts immunity after infection. In addition, the sample size of the groups with oxygen therapy requirement in our study was small.

In conclusion, our results demonstrate that the sotrovimab antibody treatment may be associated with a reduction in the requirement for oxygen therapy among high-risk patients with mild to moderate COVID-19 Omicron BA.1 and BA.2 subvariants.

## Figures and Tables

**Table 1:** Underlying conditions in patients with the COVID-19 BA.1 subvariant between the treatment and control groups *.

Variables	Treatment Group	Control Group	*p*-Value
No. of patients	642	642	
Median age (IQR), years	57 (47–63)	57 (47–63)	>0.9999
No. of males/females	391/251	391/251	>0.9999
No. (%) of patients with risk factors			
Age ≥55 years old	389 (60.6)	389 (60.6)	>0.9999
Diabetes for which medication was warranted	108 (16.8)	121 (18.8)	0.3817
Obesity with a body mass index ≥30 kg/m^2^	121 (18.8)	138 (21.5)	0.2658
Chronic kidney disease **	62 (9.7)	46 (7.2)	0.1312
Congestive heart failure ***	30 (4.7)	17 (2.6)	0.0735
Chronic obstructive pulmonary disease	86 (13.4)	93 (14.5)	0.6289
Moderate to severe asthma ****	56 (8.7)	63 (9.8)	0.5638
No. (%) of patients with COVID-19 mRNA vaccination			
Never	91 (14.2)	109 (17.0)	0.1907
One vaccination	17 (2.6)	12 (1.9)	0.4531
Two vaccinations	476 (74.1)	473 (73.7)	0.8989
Three vaccinations	58 (9.0)	48 (7.5)	0.3615
Laboratory findings, median (IQR)			
White blood cell count,/µL	4800 (3700–6000)	5000 (3900–6200)	0.4288
C-reactive protein, mg/dL	1.69 (0.70–3.25)	1.91 (0.74–3.65)	0.4398
Aspartate aminotransferase, U/L	27 (22–40)	28 (23–42)	0.6755
Alanine aminotransferase, U/L	22 (14–37)	23 (16–37)	0.7165

* Continuous values are presented as medians and interquartile ranges (IQRs) and categorical/binary values as counts and percentages. ** Estimated glomerular filtration rate, <60 mL per minute per 1.73 m^2^ of body surface area. *** New York Heart Association class II, III, or IV. **** Need for an inhaler or use of oral steroids within 1 year prior to enrollment.

**Table 2:** Clinical outcomes in patients with the COVID-19 BA.1 subvariant between the treatment and control groups *.

Variables	Treatment Group	Control Group	*p*-Value
No. of patients	642	642	
No. (%) of patients who required oxygen therapy	26 (4.0)	56 (8.7)	0.0008
Nasal cannula	20	46	
High-flow nasal cannula	4	7	
Mechanical ventilation	2	3	
No. (%) of patients who died	0	0	>0.9999

* Categorical/binary values as counts and percentages.

**Table 3:** Underlying conditions in patients with the COVID-19 BA.2 subvariant between the treatment and control groups *.

Variables	Treatment Group	Control Group	*p*-Value
No. of patients	202	202	
Median age (IQR), years	55 (46–60)	55 (46–60)	>0.9999
No. of males/females	105/97	105/97	>0.9999
No. (%) of patients with risk factors			
Age ≥ 55 years old	109 (54.0)	109 (54.0)	>0.9999
Diabetes for which medication was warranted	32 (15.8)	39 (19.3)	0.4330
Obesity with a body mass index ≥30 kg/m^2^	36 (17.8)	34 (16.8)	0.8955
Chronic kidney disease **	17 (8.4)	16 (7.9)	>0.9999
Congestive heart failure ***	9 (4.5)	7 (3.5)	>0.9999
Chronic obstructive pulmonary disease	19 (9.4)	25 (12.4)	0.4249
Moderate-to-severe asthma ****	18 (8.9)	24 (11.9)	0.4153
No. (%) of patients with COVID-19 mRNA vaccination			
Never	62 (31.0)	67 (33.2)	0.6696
One vaccination	10 (5.0)	12 (5.9)	0.8270
Two vaccinations	130 (64.4)	123 (61.4)	0.5373
Laboratory findings, median (IQR)			
White blood cell count,/µL	4900 (3800–6100)	5000 (4100–6000)	0.6953
C-reactive protein, mg/dL	1.91 (0.74–3.72)	1.88 (0.73–3.54)	0.6722
Aspartate aminotransferase, U/L	25 (21–39)	26 (21–42)	0.8002
Alanine aminotransferase, U/L	21 (14–35)	22 (16–35)	0.8211

* Continuous values are presented as medians and interquartile ranges (IQRs) and categorical/binary values as counts and percentages. ** Estimated glomerular filtration rate, <60 mL per minute per 1.73 m^2^ of body surface area. *** New York Heart Association class II, III, or IV. **** Need for an inhaler or use of oral steroids within 1 year prior to enrollment.

**Table 4:** Clinical outcomes in patients with the COVID-19 BA.2 subvariant between the treatment and control groups *.

Variables	Treatment Group	Control Group	*p*-Value
No. of patients	202	202	
No. (%) of patients who required oxygen therapy	8 (4.0)	20 (9.9)	0.0296
Nasal cannula	6	14	
High-flow nasal cannula	2	4	
Mechanical ventilation	0	2	
No. (%) of patients who died	0	0	>0.9999

* Categorical/binary values as counts and percentages.

## Data Availability

The data presented in this study are available in the article.
